# The Modulatory Role of Vitamin D in the Molecular Mechanisms of Sepsis and Infections with Focus on Viral Pathogenesis: A Narrative Review

**DOI:** 10.3390/pathogens15090894

**Published:** 2026-08-25

**Authors:** Federica Vincenzi, Maria Grazia Crobu, Stelvio Tonello, Nicole Vercellino, Elena Grossini, Paolo Ravanini, Rosalba Minisini, Lucio Boglione, Mario Pirisi, Pier Paolo Sainaghi, Carlo Smirne

**Affiliations:** 1 Department of Translational Medicine, Università del Piemonte Orientale, 28100 Novara, Italy; federica.vincenzi@uniupo.it (F.V.); stelvio.tonello@med.uniupo.it (S.T.); nicole.vercellino@uniupo.it (N.V.); elena.grossini@med.uniupo.it (E.G.); rosalba.minisini@med.uniupo.it (R.M.); lucio.boglione@uniupo.it (L.B.); mario.pirisi@med.uniupo.it (M.P.); pierpaolo.sainaghi@med.uniupo.it (P.P.S.); 2 Laboratory of Molecular Virology, Maggiore della Carità Hospital, 28100 Novara, Italy; mcrobu@cittadellasalute.to.it (M.G.C.); paolo.ravanini@maggioreosp.novara.it (P.R.); 3 Clinical Biochemistry Laboratory, Department of Laboratory Medicine, City of Health and Science University Hospital, 10126 Turin, Italy; 4 Interdisciplinary Research Centre of Autoimmune Diseases (IRCAD), 28100 Novara, Italy; 5 Internal Medicine Unit, San Giovanni Bosco Hospital, ASL Città di Torino, 10154 Turin, Italy

**Keywords:** vitamin D, viral infections, sepsis, cholecalciferol, calcitriol, hepatitis C virus, hepatitis B virus, SARS-CoV-2, Epstein–Barr virus, influenza virus

## Abstract

Viral infections are a widely recognized cause of mortality. Recently it has been demonstrated that vitamin D plays an important immuno-modulatory role in both innate and adaptive immune responses against infections, by reducing excessive inflammation and enhancing defense mechanisms. Specifically, exacerbation of infections may cause sepsis, characterized by a dysregulated immune response with hyperinflammation and immune exhaustion. Vitamin D can modulate these processes through various mechanisms, such as enhancing antiviral protection, promoting anti-inflammatory responses, protecting endothelial barriers, and modulating T-cells. This has been demonstrated for various viral infections, including influenza viruses, respiratory syncytial virus, and severe acute respiratory syndrome coronavirus. Although vitamin D deficiency has been associated with increased susceptibility to viral infections and immune cells of infected patients are highly responsive to vitamin D, the clinical benefits of its supplementation to vitamin D-deficient infected individuals are uncertain and, most importantly, direct evidence linking vitamin D to viral sepsis specifically remains particularly limited, with most mechanistic inference extrapolated from non-septic viral infection and bacterial sepsis literature. Based on these assumptions, this review will outline the present understanding about vitamin D regulatory effects on immune system and the possible connection between its serum levels and viral infections, while also addressing controversial issues.

## 1. Introduction

Vitamin D is a prohormone produced in the skin after ultraviolet (UV)-B light exposure. Deficiency and insufficiency of this factor are widely recognized issues, particularly in elderly patients; moreover, an increasing body of knowledge on the immunomodulatory functions of vitamin D has gradually been built up. Therefore, the contribution of this compound in the pathogenesis of several diseases has been widely studied. In particular, vitamin D insufficiency/deficiency may play a role in immune system weakening and disease worsening, especially in subjects with an already compromised immune system. In this context, a dysregulated immune response may have an impact in increased susceptibility to infections, including those of viral origin. To note, severe viral infections may result in sepsis, defined as a life-threatening condition characterized by organ dysfunction mediated by dysregulated host response to infection, while septic shock outlines cellular, metabolic, and circulatory abnormalities related to increased risk of mortality [1].

Sepsis etiology can more commonly be ascribed to bacterial (or fungal) infections; this probably explains why viral sepsis is frequently underdiagnosed and viruses are not routinely analyzed in clinical practice. The definition of viral sepsis is still in debate, because of the complex pathological interplay between viruses and immune system. Frequently, patients infected by chronic viruses, such as hepatitis B virus (HBV) and hepatitis C virus (HCV), or latent viruses, including herpes-simplex virus (HSV) or human immunodeficiency virus (HIV), show Sequential Organ Failure Assessment (SOFA) scores fulfilling the Sepsis-3 criteria. However, due to the late onset of symptoms and organ damage, this type of measurement usually indicates terminal disease rather than sepsis. Hence, viral sepsis can be better diagnosed in presence of an acute viral infection leading to organ dysfunction, ruling out chronic or latent infections [2]. Although, most evidence supports the immunomodulatory effects of vitamin D during viral infections and sepsis, the clinical effectiveness of vitamin D supplementation remains controversial. Indeed, several experimental and observational studies associate vitamin D deficiency with increased susceptibility to infections and poorer clinical outcomes, while clinical trials show heterogeneous results, highlighting the difficulties in translating experimental research into clinical practice. To thoroughly comprehend the potential contribution of vitamin D in infectious diseases, it is crucial to analyze its molecular biology and immunomodulatory functions within the immune system. In this review we will describe the mechanisms by which vitamin D can modulate the inflammatory signaling responsible for the pathophysiology of most infections with particular reference to those of viral origin, providing observations regarding its biological effects and immunomodulatory role.

It should be noted that this paper was conceived as a narrative review of the current literature regarding the role of vitamin D in infections, with a special focus on viral infections and viral sepsis. Literature search was performed using keyword strings including “vitamin D” OR “calcitriol” OR "1,25-dihydroxyvitamin D3” OR “cholecalciferol OR “vitamin D receptor"” AND (“sepsis” OR “infection” OR “viral sepsis” OR “viral infection”) AND (“viral pathogenesis” OR “virus” OR "molecular mechanisms" OR “viral replication” OR “SARS-CoV-2” OR “influenza”) AND (“cytokines” OR "immune modulation" OR “immunity”). Studies were selected based on the relevance to the molecular mechanisms linked to infections and sepsis, emphasizing the experimental, translational and clinical evidence.

## 2. Vitamin D Biology in Immune Regulation

### 2.1. Vitamin D Synthesis, Intermediates in the Body and Molecular Signaling

Vitamin D is a fat-soluble steroid, and the term refers to both plant-derived vitamin D_2_ (ergocalciferol), introduced primarily through the diet from plants, including yeast and fungi, and vitamin D_3_ (cholecalciferol), produced in the skin or derived from animal sources [3].

In humans, cholecalciferol, the inactive form, is synthetized in the skin after UV-B light exposure starting from 7-dehydrocholesterol. Cholecalciferol is then carried to the liver through the vitamin D binding protein (VDBP), where it is transformed by the enzyme 25-hydroxylase to the most abundant circulating form, calcidiol. Due to its long half-life and the elevated circulating levels, calcidiol is the most solid indicator of vitamin D status. Currently, a consensus has not been achieved about the levels of calcidiol suggestive of sufficiency, insufficiency and deficiency. Indicatively, calcidiol levels of 30–150 ng/mL can represent sufficiency, while 21–29 ng/mL are suggestive of insufficiency. Therefore, those with circulating calcidiol levels below 20 ng/mL are considered deficient [4,5].

Vitamin D deficiency is a major burden among the global population. The prime cause is reduced sunlight exposure, which varies according to latitude, altitude, season, sunscreen use, age, skin color and clothing habits. Moreover, malabsorption due to gastrointestinal disorders or other conditions, as well as the use of some medications, may increase the risk of deficiency [6].

In the kidney, calcidiol is then hydroxylated by 1α-hydroxylase to calcitriol, the functionally active form of vitamin D [7]. To avoid toxic accumulation, calcitriol can downregulate itself through a negative feedback, inducing 24-hydroxylase production, which hydroxylates both calcitriol and calcidiol in position C-23 and C-24, favoring their excretion [8]. Calcitriol exerts its genomic functions by interacting with the cytosolic vitamin D receptor (VDR), which is further phosphorylated and forms a complex with the retinoid-X-receptor (RXR). This ligand-receptor complex translocates to the nucleus, where it interacts with vitamin D response elements (VDREs), regulating gene expression. Moreover, calcitriol can also exert non-genomic functions by binding to membrane VDR and regulating several pathways according to the cell type. For example, it is known that activation of phospholipase C (PLC), phospholipase A2 (PLA2), and phosphatidylinositol-3 kinase (PI3K), along with the generation of secondary messengers, can induce intracellular signaling by triggering protein kinase A (PKA), protein kinase C (PKC), and mitogen-activated protein kinases (MAPK) [9]. The most known function of calcitriol consists in the regulation of calcium homeostasis, thus contributing to bone health. However, various non-skeletal effects have been increasingly reported after discovering VDR presence in several other tissues including prostate, brain, breast, colon, pancreas and immune cells [10]. This last point in particular emphasizes the importance of vitamin D in numerous biological functions, encompassing the regulation of both innate and adaptive immunity, and highlights the potential immunomodulatory role of calcitriol in the context of infections.

In the following section we will thus analyze the effects of calcitriol on the immune system, concentrating on the various responses to infectious agents.

### 2.2. Immunomodulatory Functions of Vitamin D

The widely recognized immunomodulatory role of calcitriol has led researchers to study its impact in several pathologies, including autoimmune diseases, infections and sepsis. Specifically, this section will outline the common immunomodulatory functions of vitamin D, providing the mechanistic framework for comprehending its role in infections and sepsis, with a particular focus on those of viral origin.

It is well known that calcitriol regulates the function of monocytes/macrophages, dendritic cells, and B and T cells, thus contributing to a balanced immune response and limiting excessive inflammation and oxidative stress [11]. In particular, both VDR and 1α-hydroxylase are expressed in immune cells, evidencing their capacity to metabolize calcidiol. For instance, it has been reported that the activation of toll-like receptors (TLRs) by *Mycobacterium tuberculosis* (*Mtb*) in human macrophages induces the production of 1α-hydroxylase, therefore leading to calcitriol synthesis and activation of VDR signaling [12]. The kidney levels of 1α-hydroxylase are in turn regulated by parathyroid hormone (PTH), while activation of this enzyme in extrarenal sites, including monocytes/macrophages, is mediated by calcidiol blood levels, along with different inflammatory molecules, such as tumor necrosis factor (TNF)-α, interleukin (IL)-1 and interferon (IFN)-γ [13].

Moreover, calcitriol can downregulate dendritic cells (DCs) differentiation and IL-12 production, therefore decreasing T cell stimulation. In this way, calcitriol can negatively regulate cluster differentiation (CD)4^+^ T helper (Th)1 and Th17 responses, characterized by elevated IFN-γ and IL-2 production, inducing a switch to Th2 and regulatory T (Treg) cells suppressive phenotypes [11,14]. Indeed, calcitriol-VDR-RXR complex can recognize a silencer region of the IFN-γ gene, inhibiting its transcription. In addition, the complex can bind nuclear factor of activated T-cells (NFAT) on IL-2 promoter, blocking the formation of NFAT/activator protein-1 (AP-1) complex and inhibiting IL-2 production [7]. Modulation of the inflammatory milieu is also mediated by the regulation of nuclear factor kappa-light-chain-enhancer of activated B cells (NF-κB) and MAPK [9]. These findings underline the mechanisms by which calcitriol dampens the pro-inflammatory response, while enhancing the production of anti-inflammatory mediators.

Finally, calcitriol is also able to induce autophagy and gap proteins production, strengthening the integrity of epithelial cells and averting viral entry. Interestingly, calcitriol can also induce the production of neutralizing antibodies and antibacterial molecules, essential for the resolution of infections [15].

### 2.3. Vitamin D-Mediated Antimicrobial Mechanisms

Evidence has been collected regarding the effects of vitamin D on macrophage-mediated antimicrobial response, primarily in the context of bacterial infections. Studies on *Mtb*-stimulated macrophages showed that both calcidiol and calcitriol can activate the gene encoding for the antimicrobial peptide cathelicidin (LL37), resulting in the killing of *Mtb* [16]. Cathelicidin antiviral properties rely instead on the suppression of viral entry, replication and assembly, along with the activation of the immune response. Therefore, research is currently focusing on the production of efficient cathelicidin derivatives to treat viral infections [17,18]. Moreover, it is worth noting that in human promonocytic leukemia cells (U937) stimulated with calcitriol, cathelicidin induction was demonstrated after 1 to 3 h, and was maintained throughout the 5 days of treatment in a dose-dependent manner. Interestingly, inhibition of protein synthesis was irrelevant for the expression of cathelicidin gene, showing that the latter one is a direct target of VDR, and is not triggered by the synthesis of other VDR-induced transcription factors [19]. Supporting this finding, Wang et.al reported the presence of VDRE consensus sequences in the promoters of cathelicidin and defensin-β2 (defB2) genes, suggesting the capacity of calcitriol to elicit the expression of these two powerful antimicrobial peptides [20]. In contrast, Liu et al. showed that calcitriol alone cannot induce the expression of defB2 mRNA. Specifically, while triggering of the TLR 1/2 signaling resulted in the activation of the VDR pathway, leading to cathelicidin expression; TLR-mediated expression of defB2 required an interplay between IL-1β and VDR pathways. Moreover, the presence of one VDRE and two NF-κB response elements in the promoter of defB2 gene suggested the requirement of NF-κΒ for optimal gene activation. Indeed, transfection of NF-κB p65—the main IL-1β-induced subunit—into human-derived monocytes enhanced defB2 expression only after VDR activation, highlighting the interplay of these two pathways [21].

The antibacterial properties of vitamin D are not exclusively related to the activation of TLR. Indeed, calcitriol can induce the production of nucleotide-binding and oligomerization domain (NOD)-2, an intracellular pathogen-recognition receptor (PRR) able to bind to muramyl dipeptide (MDP), a membrane product of both Gram-negative and Gram-positive bacteria [22]. In fact, after phagocytosis and phagolysosome digestion of the pathogen, intracellular binding of MDP to NOD2 can activate the NF-κB pathway, inducing the production of defB2 and cathelicidin [22,23].

Calcitriol also regulates the expression of hepcidin antibacterial protein (HAMP), whose main function is to suppress ferroportin-mediated export of iron. By binding to VDRE sequences within the HAMP gene promoter, calcitriol downregulates hepcidin expression and restores the iron-exporting activity of ferroportin, thereby reducing intracellular iron availability and limiting microbial growth. Indeed, iron represents an essential nutrient for the growth and replication of most microorganisms; therefore, intracellular iron accumulation may favor pathogen proliferation, including *Mtb*. Consequently, limiting intracellular iron availability may contribute to pathogen control and resolution of infection [24]. Hepcidin is activated by several pathogens-associated molecular patterns (PAMPs) and inflammatory stimuli. With specific regard to viral diseases, hepcidin production can for instance be stimulated by various pro-inflammatory stimuli, including IL-1β, IL-6 and TNF-α, which are universally activated during these processes. Indeed, hepcidin levels result to be increased during several viral infections like those due to severe acute respiratory syndrome coronavirus (SARS-CoV-2) or Epstein–Barr virus (EBV). In cases of chronic infections mediated by HCV or HBV, these elevated levels of hepcidin lead also to iron accumulation in the liver, thereby increasing oxidative stress and inducing organ damage, with enhanced risk of hepatocellular carcinoma development [25]. Finally, hepcidin has been identified as an enhancer of HIV replication, since iron is an essential component for this process [26].

These findings shed light on how different mechanisms are involved in calcitriol-mediated antimicrobial responses, highlighting the importance of pathogen–TLR interaction in the eradication of infectious agents. In addition to the aforementioned direct antimicrobial activities, it is also involved in many other pathways that interact with host immune responses, contributing to the regulation of processes such as autophagy induction, epithelial barrier integrity and inflammatory signaling. For what concerns autophagy—a mechanism essential to maintain homeostasis in several physiological and pathological processes such as cancer and infections—studies have shown that calcitriol can promote autophagosome-lysosome fusion in adenocarcinoma human alveolar basal epithelial cells (A549) infected with influenza A virus (IAV) [27]. Consistent with this, Caseti et al. found that HIV patients with hypovitaminosis D exhibited lower expression of autophagy markers on peripheral blood mononuclear cells (PBMCs), regardless of whether they were receiving antiviral therapy [28]. Calcitriol can also regulate autophagy through the generation of antimicrobial peptides. Indeed, calcitriol-induced antimicrobial peptides such as cathelicidin and def2B have been shown to augment autophagosome-phagosome fusion, facilitating intracellular pathogen clearance [29]. Beyond the induction of antimicrobial peptides and autophagic pathways, calcitriol also contributes to host defense by sustaining epithelial and endothelial barrier integrity, which represents a critical determinant of infection susceptibility and consequent disease severity. Specifically, calcitriol enhances the synthesis of tight junction proteins, such as occludin and claudins, within the skin, thereby maintaining epithelial barrier function and preventing the infiltration of pathogens [30].

Importantly, although, as previously mentioned, many of these complex mechanisms have been explored in the context of bacterial infections, there is growing evidence that they can also contribute to the early stages of viral infections. Therefore, understanding how viruses interact with these pathways is essential to define the molecular basis of antiviral immune responses, as next section will depict.

## 3. Molecular Mechanisms of Antiviral Response

### 3.1. Innate Immune Sensing of Viral Pathogens

**Figure 1:**
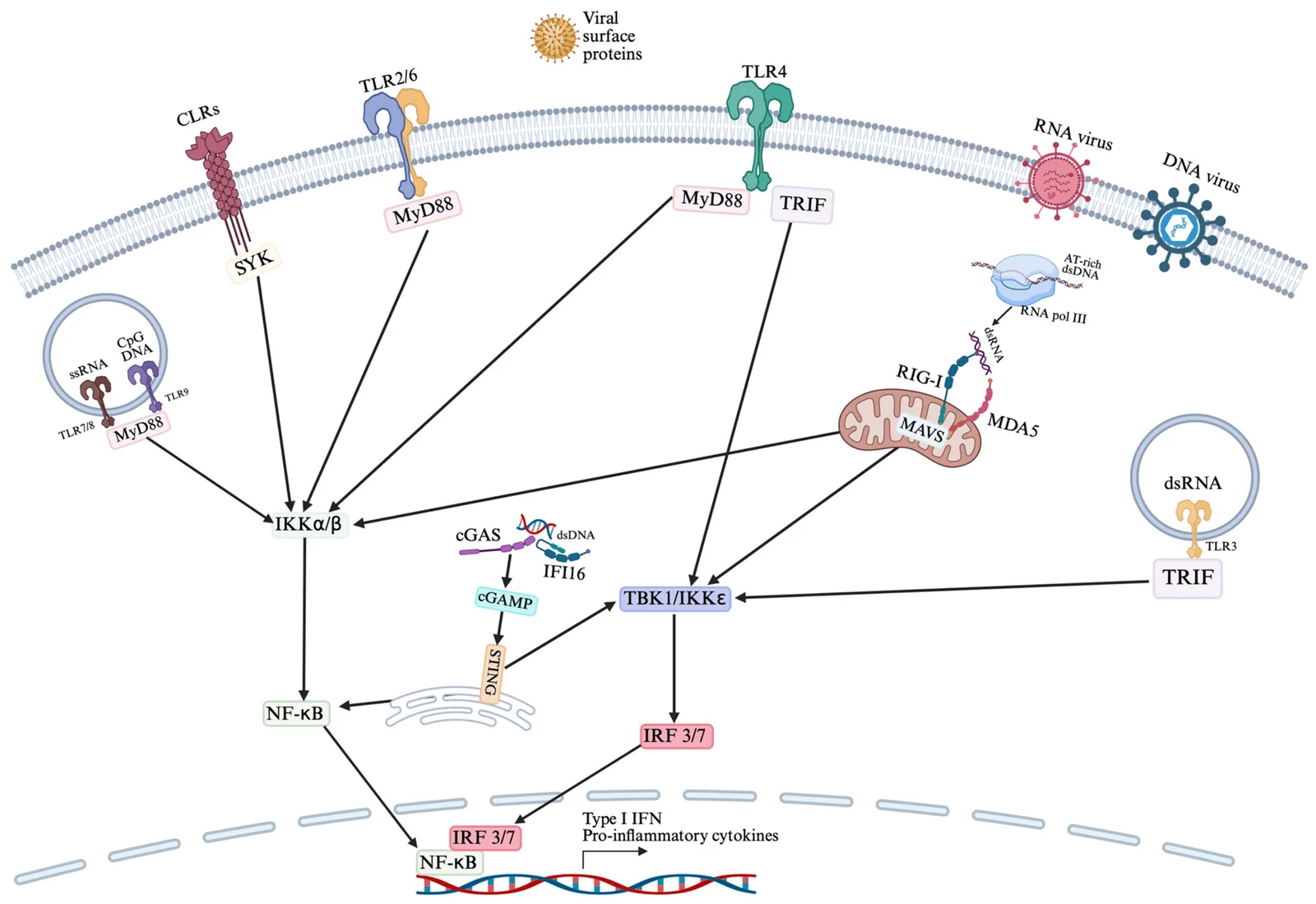
Representation of PRRs and the major downstream pathways involved in antiviral innate immune response. Created in BioRender. Minisini, R. (2026) https://BioRender.com/99c9r0a (accessed on 31 May 2026).

Viral infections represent a complex host–pathogen interaction in which both immune and non-immune cells can be targeted, leading to direct tissue injury and triggering innate and adaptive immune responses. However, viruses activate immune response differently from bacteria and fungi. Following the entry into host cells, PAMPs, such as viral nucleic acids and structural proteins, are recognized by PRRs including TLRs, C-type lectin receptors (CLRs), NOD-like receptors (NLRs), and intracellular sensors including retinoic acid-inducible gene-I (RIG-I)-like receptors (RLRs), absent in melanoma 2 (AIM2), and cyclic guanosine monophosphate adenosine monophosphate synthase (cGAS). Additionally, damage-associated molecular patterns (DAMPs) released by infected or damaged cells, comprising host DNA and proteins, further contribute to immune activation (Figure 1) [31].

As summarized in Figure 1, receptor engagement initiates intracellular signaling pathways that ultimately converge on a limited number of downstream signaling mechanisms, mainly NF-κB-mediated inflammatory responses and interferon regulatory factors (IRF3/7)-dependent type I IFN production. More in detail, viral coat proteins are recognized either by surface CLRs or TLRs, like TLR2/6 and TLR4. Binding of viral antigens to CLRs engages spleen tyrosine kinase (SYK), which in turn leads to inhibitory κB kinase (IKK)α/β complex resulting in NF-κB activation. TLR2/6 heterodimer and TLR4 downstream signaling require the activation of the toll/interleukin-1 receptor (TIR) domain of myeloid differentiation primary response protein 88 (MyD88), as well as of TIR domain-containing adapter-inducing interferon-β (TRIF) for what concerns TLR4. MyD88 leads to IKKα/β complex triggering, with subsequent stimulation of NF-κB. However, if the involvement of these receptors results in IL-10 production it may favor viruses by inducing viral immune evasion. Downstream of TLR4, TRIF can induce the activation of TANK-binding kinase (TBK)1/IKKε which consequently stimulates interferon regulatory factors (IRF)3/7.

Sensing of viral nucleic acids is quite complex and can be mediated by receptors both on endosomes and in the cytoplasm. In particular, the endosomal receptors TLR7/8 heterodimer, TLR9 and TLR3 recognize single strand RNA (ssRNA), cytosine-phosphate-guanine (CpG) DNA and double strand DNA (dsDNA), respectively. TLR7/8 heterodimer and TLR9 signal through the MyD88 pathway, leading to NF-κB activation, while TLR3 activates the TRIF pathway. Viral dsDNA is sensed in the cytosol by cyclic guanosine monophosphate adenosine monophosphate (GMP-AMP) synthase (cGAS) and the accessor protein interferon gamma inducible protein 16 (IFI16), which both help cGAS to synthetize cyclic GMP-AMP (cGAMP). cGAMP in turn can bind and switch on stimulator of interferon genes (STING) which is capable of activating both NF-κB and IRF3/7. Another mechanism of cytosolic viral sensing is mediated by RNA polymerase III (RNA pol III) which transcribes AT-rich DNA to double strand RNA (dsRNA). dsRNA is then sensed by the RLRs, RIG-I and melanoma differentiation-associated gene 5 (MDA-5) which, through the adaptor mitochondrial antiviral-signaling protein (MAVS), activate both NF-κB and IRF3/7. The recruitment of NF-κB and IRF3/7 ultimately leads to their translocation to the nucleus, where they act as transcription factors for inflammatory cytokines and type I IFNs and promote a predominantly antiviral state. The latter condition is achieved also by influencing the survival, maturation, and migration of immune cells to the site of infection, as well as by activating adaptive immunity [32]. In this context, particularly relevant is the type I IFN response—discussed in the next section—which represents the initial and most essential mediator of the antiviral response.

### 3.2. Interferon-Mediated Antiviral Responses

Type I IFNs constitute the major players of antiviral response, orchestrating a complex transcriptional program that limits viral replication and shapes downstream immune responses, by activating a signaling cascade that promotes phosphorylation and nuclear translocation of transcription factors responsible for regulating IFN-stimulated genes (ISGs) [33]. Upon engagement with IFNα receptor 1 (IFNAR1)/IFNAR2 heterodimer, type I IFNs initiate the activation of the coupled Janus kinase 1 (JAK1) and tyrosine kinase 2 (TYK2). This activation leads to the phosphorylation of IFNAR1, subsequently facilitating the recruitment and phosphorylation of signal transducers and activators of transcription (STAT)1 and 2. These phosphorylated proteins combine with IRF9, to create a complex termed IFN-stimulated gene factor 3 (ISGF3). ISGF3 is subsequently translocated into the cell nucleus, where it binds to IFN-stimulated response elements (ISREs), promoting the transcription of ISGs [34]. Additionally, type I IFNs possess the capability to initiate subsequent signaling cascades orchestrated by MAPK and PI3K pathways, eventually regulating ISGs expression [35].

ISGs encode a wide range of antiviral effectors, which perpetuate the strong response against viruses; some act as positive regulators that enhance innate immune response, and other as negative controllers responsible for terminating the signaling [36]. Effector genes include IFN-stimulated protein of 15 kDa (ISG15), an ubiquitin homologue, which modulates protein stability and viral assembly; myxovirus resistance (Mx) protein, which induces viral nucleocapsid degradation; 2′,5′-oligoadenylate synthetase (OAS), which activates ribonuclease L (RNaseL) leading to viral RNA degradation; and protein kinase R (PKR), which inhibits viral protein translation [34]. Moreover, ISGs include restriction factors which can impair the release of progeny virions or capsid formation and DNA degradation, ensuring a coordinated defense against viral infections [37]. Importantly, ISGs comprise negative regulators of IFN signaling, such as suppressor of cytokine signaling (SOCS), which inhibit STAT binding to IFN receptor, and ubiquitin-specific protease 18 (USP18), which prevents JAK association to the receptor, facilitating restoration of immune homeostasis [38].

In addition to type I IFNs, type III IFNs also play a critical role in localized antiviral responses, particularly acting on epithelial barriers. For instance, respiratory epithelial cells release IFN-III in response to IAV, respiratory syncytial virus (RSV) and other infections. Despite the differences in cell specific expression of IFN-I and IFN-III receptors, they stimulate a similar range of ISGs, which reinforce the immune response to viruses [39].

Ultimately, IFN signaling elicits several immunological functions, including promotion of antigen presentation, production of inflammatory mediators, and modulation of adaptive immune cells. Specifically, beyond their direct antiviral properties, IFNs play an essential role in linking innate and adaptive immunity, ultimately determining effective viral clearance and disease outcome. However, while IFNs are crucial for viral control, dysregulated IFN response may result in tissue damage, chronic infections, inflammatory and autoimmune diseases. Therefore, IFN signaling is tightly regulated by several feedback mechanisms to ensure effective antiviral response while limiting hyperinflammation and tissue toxicity [40].

### 3.3. Adaptive Immune Responses to Viral Infections

The detection of viral components via PRRs on antigen presenting cells (APCs), like DCs, can initiate an adaptive immune response specifically targeting the virus. Viral clearance is predominantly facilitated by cytotoxic CD8^+^ T lymphocytes, whose activation is driven by IFNs, IL-12 and cross-presentation of viral antigens by DCs [41]. However, DCs also mediate the activation of different CD4^+^ Th cells, which are crucial for regulating immune responses, including activation of tissue resident macrophages, modulation of effector T cells, and enhancement of antibody production from B cells [42]. Importantly, the major mediators of viral infections are plasmacytoid DCs (pDCs) which are capable to generate substantial quantities of type I IFNs, thereby contributing to the various steps of antiviral response, including the activation of natural killer (NK) and T cells. While pDCs contribute, to some extent, to T cell activation, it is primary conventional DCs that are responsible for presenting antigens to naïve T cells [43].

CD8^+^ T cells are an important population responsible for viral clearance. Specifically, virus-specific peptides presented by major histocompatibility complex (MHC)-I are recognized by T cell receptor (TCR). Upon engagement with the antigen, CD8^+^ T cells expand and differentiate to effector T cells, capable of producing antiviral cytokines, including IFNs, as well as exert cytotoxic activities through granzymes and perforin production. After the acute phase of viral infections, T cells can persist as memory T cells, able to exert a secondary immune response upon reencountering the same viral pathogen [44]. However, chronic exposure to viral antigens induces T cell exhaustion, distinguished by diminished proliferation and secretion of cytotoxic cytokines. For instance, in chronic HIV infection, cytotoxic CD8^+^ T cells progressively lose their function and start expressing inhibitory receptors including cytotoxic T lymphocyte associated protein-4 (CTLA-4), programmed cell death-1 (PD-1), lymphocyte activation gene 3 (LAG-3) and T cell immunoglobulin domain and mucin domain 3 (TIM-3), potentially leading to functional impairment and apoptosis [45].

In contrast, CD4^+^ Th cells recognize virus specific antigens presented by MHC-II. Depending on the affinity of the TCR/MHC-II interaction and the cytokine/chemokine milieu, CD4^+^ T cells differentiate in several subpopulations harboring distinct functions. In particular, Th1 cells release IFN-γ, TNF-α and IL-2, thereby activating NK and CD8^+^ T cells. Conversely, Th2 cells primarily govern antibody-mediated immunity and influence antibody class-switching through the secretion of IL-5 and IL-10. Furthermore, T follicular helper (Tfh) cells secrete IL-21, thus coordinating B cell response and CD8^+^ T cell function; while Th17 cells, by releasing IL-17, mediate the pro-inflammatory response and promote inflammatory cell infiltration to the infected site [46]. Lastly, T regulatory (Tregs) cells are an immunosuppressive subtype of CD4^+^ T cells, specialized in maintaining immune homeostasis and self-tolerance [47]. These cells can directly kill T effector cells through granzyme/perforin or suppress APCs by downregulating CD80/CD86 expression on APCs via CTLA-4 [48].

However, imbalance between Treg and Th17 cells, defined by decreased Tregs and elevated Th17 cells, may impair viral clearance. For instance, increased Th17/Treg ratio has been reported in SARS-CoV-2 infected patients with worst clinical outcome compared to improved cases [49]. Moreover, in RSV infection, Th17-mediated recruitment of neutrophil on the airway as well as mucus production may impair viral clearance [50].

## 4. The Interplay Between Vitamin D and Viral Infections

### 4.1. Vitamin D as a Modulator of Antiviral Immune Responses

Vitamin D may play a role in directing the immune responses to viruses so far described—with particular reference to those of the innate type—through the modulation of pathogen recognition, cytokine secretion and oxidative stress. These mechanisms are pertinent in the context of viral infections, where disease severity and progression toward systemic immune dysregulation mainly rely on the balance between antiviral defense and excessive inflammation. This is particularly true for respiratory viral diseases, like IAV, SARS-CoV-2 and RSV, as well as for HIV, EBV, HCV and HBV infections. In such cases, vitamin D immunomodulation mainly relies on its ability to influence specific transcription factors crucial for initiating the synthesis of antimicrobial peptides, lowering of inflammation and triggering of autophagy, as previously mentioned.

Respiratory epithelial cells exhibit a pronounced susceptibility to vitamin D. It has been established that bronchial epithelial cells express CYP27B1, the enzyme responsible for the conversion of calcidiol to calcitriol, and its expression is proven to increase following RSV infection [51]. Moreover, both calcidiol and calcitriol treatments induce the expression of CYP24A1, CD14 and cathelicidin in human bronchial epithelial cells, with cathelicidin also detected at protein level in cell supernatant [52]. Beyond its antimicrobial activity, cathelicidin can enhance TLR3-mediated signaling in bronchial epithelial cells, easing viral sensing and innate immune signaling [53].

Vitamin D also participates in the regulation of inflammation generated by viral infections. In RSV-infected airway epithelial cells, calcitriol promotes IκBα expression, inhibiting NF-κB activation and decreasing the production of IFN-β1, C-X-C motif chemokine ligand (CXCL)10 and other inflammatory mediators, without increasing viral replication [54]. This evidence suggests that calcitriol may attenuate excessive inflammatory response while preserving antiviral defense mechanisms.

Similarly, dysregulated innate immune activation and impaired type I IFN responses represent key hallmarks of SARS-CoV-2 infection. In this context, vitamin D supplementation has been related with amplified IFNα/β signaling and augmented expression of RIG-I/MDA5 and JAK/STAT pathways along with several antiviral ISGs, suggesting a potential role for vitamin D in restoring antiviral innate immune pathways during Coronavirus Disease 19 (COVID-19) [55]. Moreover, it has been established that vitamin D regulates antiviral response against SARS-CoV-2 through antimicrobial peptides, which can adhere to both the spike protein and its receptor angiotensin-converting enzyme-2 (ACE2), therefore protecting from viral infection [56].

Another mechanism through which vitamin D may influence antiviral immunity is depicted by the regulation of autophagy, a process involved in intracellular pathogen clearance and maintenance of cellular homeostasis. Calcitriol increases microtubule-associated protein 1 light chain 3 beta (LC3B)-II protein levels and inhibits viral replication in HIV and/or *Mtb*-infected monocyte-derived macrophage (MDM) cells, supporting the role of vitamin D-induced autophagy in pathogen control [57,58]. Interestingly, silencing of cathelicidin significantly reduces calcitriol-mediated autophagy and suppression of HIV replication, indicating that antimicrobial peptides contribute to the interconnection between vitamin D signaling, autophagy and antiviral response [58]. The pro-autophagic effects of calcitriol have also been established in IAV-infected A549 cells, where calcitriol reduces apoptosis and enhances autophagosome-lysosome fusion, thereby restoring autophagic flux and contributing to viral clearance [27]. Supporting these findings, circulating vitamin D levels have been directly correlated with autophagy-related proteins, including autophagy related 7 (ATG7) and beclin 1 (BECN1) levels in SARS-CoV-2 infected patients [59]. Since mitochondrial dysfunction and oxidative stress are key drivers of tissue damage during severe viral infections, vitamin D-mediated regulation of autophagy may thus represent an important mechanism for limiting virus-induced cellular injury.

In addition to regulating inflammatory signals and autophagy, calcitriol has been proven to exert antioxidant effects by modulating pathways involved in reactive oxygen species (ROS) production and cellular antioxidant defense. Physiological concentrations of calcidiol have been documented to augment the expression of nuclear factor erythroid-2 (Nf-E2)-related factor 2 (Nrf2), a major regulator of antioxidant responses inversely correlated with ROS production [60]. In addition, VDR signaling regulates the transcription of genes that modulate mitochondrial respiration and adenosine triphosphate (ATP) synthesis. Interestingly, VDR activation has been related to mitochondrial activation and maintenance of cellular homeostasis, whereas impaired VDR signaling could result in mitochondrial dysfunction and augmented oxidative stress. Although physiological ROS production is required for antiviral defense, excessive ROS generation contributes to epithelial and endothelial damage, amplification of inflammatory pathways, and development of tissue damage during severe viral infections. In this regard, the antioxidant effects mediated by vitamin D may diminish SARS-CoV-2 associated complications, through the limitation of oxidative stress and inflammatory response [61]. Similarly, hypovitaminosis D has been related to elevated oxidative stress markers in patients affected by HCV, further supporting a connection between vitamin D status, redox imbalance and disease severity [62].

In addition to all the processes previously cited, gene expression analyses may be particularly useful for identifying specific genetic signatures and gaining a deeper understanding of the antiviral mechanisms of vitamin D. Indeed, transcriptomic analyses such as bulk and single-cell RNA sequencing (scRNA-seq) revealed that vitamin D acts as an immune system rheostat, inducing antimicrobial and anti-inflammatory proteins, resolving inflammation by converting pro-inflammatory Th1 cells (which, as above reported, secrete IFN-γ) into regulatory cells, and modulating IFN signaling pathways to prevent overactive immune responses. In this respect, advanced transcriptomics have provided high-resolution insights into how the active form of vitamin D (1,25-dihydroxyvitamin D3) and the VDR alter many of the cellular states described in the previous sections. Specifically, scRNA-seq revealed that vitamin D actively upregulates PRRs and antimicrobial peptides, such as cathelicidin and defensins, which target viral envelopes and inhibit replication. Moreover, sequencing studies showed that vitamin D enhances host responses to viruses by modulating type I IFN pathways, enhancing RIG-1/MDA-5 and Jak/STAT signal transduction pathways, and rapidly triggering the transcription of ISGs. All these mechanisms ultimately help create the conditions for establishing an antiviral state in respiratory and epithelial cells. Bulk RNA sequencing of virally infected cells also documented the ability of vitamin D to suppress harmful pro-inflammatory pathways (such as the TNF-induced NF-κB signaling cascade) and to downregulate inflammatory cytokines (such as IL-6 and TNF-α), while upregulating IL-10 (which protects host tissues from excessive damage during viral clearance). Similarly, transcriptomics studies revealed that vitamin D can mediate the transition of the pro-inflammatory IFN-γ -producing Th1 cells toward more suppressive, regulatory phenotypes [63,64].

Gene expression studies also showed that vitamin D supplementation inhibits TLR signaling by reducing the expression of CD14, the co-receptor of TLR4, and triggering receptor expressed on myeloid cells 1 (TREM1), along with adaptor proteins and transcription factors [63]. These regulatory effects have been also identified in PBMCs stimulated with lipopolysaccharide (LPS), where vitamin D modulates immune-specific genes involved in proliferation and phagocytic capacity [64]. Moreover, an immune regulatory transcriptomic signature in response to vitamin D exposure has been identified both in vivo and in vitro (THP-1 cells and PBMCs), which includes CD14, CD93, cathelicidin, CCAAT enhancer binding protein beta (CEBPB), MAPK13, leukocyte immunoglobulin like receptor B4 (LILRB4) and TREM1, all genes involved in immune function [65]. Additionally, it has been reported that supplementation with this compound modulates epigenomic remodeling, thus contributing to the transcriptional regulation of pathways involved in innate immunity and IFN signaling. More in detail, scRNA-seq highlighted key dynamic changes in the epigenetic landscape of CD4^+^ T-cells. with the generation of super-enhancers and the recruitment of several transcription factors, notably Jun proto-oncogene, AP-1 transcription factor subunit (c-JUN), STAT3 and BTB domain and CNC homolog 2 (BACH2), which together with the VDR—intrinsically expressed by activated T lymphocytes—shape the transcriptional response to vitamin D [66]. While taking all these considerations into account, it must be said that all this transcriptomic evidence derives mainly from non-septic, non-sepsis-specific models, and has not yet been generated in a viral sepsis context.

Despite these significant limitations, taken together these observations indicate that vitamin D may support antiviral immunity through several integrated mechanisms, including regulation of inflammation, enhancement of antimicrobial peptide secretion, modulation of autophagy, and preservation of redox homeostasis, along with a deep transcriptomic regulation of immune-related pathways. Specifically, the abovementioned molecular mechanisms might provide the biological rationale supporting a potential role of vitamin D in the development of viral disorders too. Based on these pathophysiological premises, numerous clinical investigations have examined the impact of vitamin D supplementation on viral disease susceptibility, severity and overall clinical outcomes. However, as stated above, translating mechanistic findings into clinical benefits remains challenging to date, also because clinical evidence is heterogeneous and highly influenced by vitamin D status, supplementation regimen and timing.

Thus, given the complexity of the issues involved, in the following sections we will try to summarize the current clinical evidence regarding the role of vitamin D in the context of infectious diseases, with focus on those of viral origin, considering both observational and interventional studies.

### 4.2. Clinical Evidence of Vitamin D in Viral Infections

Despite the extensive evidence concerning vitamin D immunomodulatory and antiviral properties, the actual clinical effectiveness of vitamin D supplementation remains, as above reported, a matter of debate.

Numerous observational studies have indicated that insufficient vitamin D levels may correlate with increased vulnerability to viral infections, greater disease severity and worse prognosis. However, these data should be interpreted with caution, since observational evidence alone is not sufficient to establish causality. Indeed, vitamin D may also be a marker of disease severity rather than a well-established risk factor. Nevertheless, interventional studies results are highly heterogeneous, probably due to discrepancies in design, dosage and timing of supplementation, baseline vitamin D levels and target population. Taking these considerations into account, in the following sections we will address the current knowledge regarding vitamin D and its supplementation within the framework of the most common respiratory viral infections and non-respiratory chronic viral infections.

#### 4.2.1. Acute Respiratory Viral Infections

Respiratory viruses represent the most examined group of pathogens concerning vitamin D supplementation. Numerous studies have investigated the connection between vitamin D levels and the susceptibility to influenza virus, RSV and SARS-CoV-2 infections (Table 1), along with the potential contribution of vitamin D supplementation (Table 2).

Vitamin D deficiency has been associated with increased likelihood of upper respiratory tract infections, pneumonia and poorer clinical outcomes, sustaining the hypothesis that vitamin D might contribute to the regulation of immune homeostasis within the respiratory system. Indeed, a meta-analysis conducted by Martineau et al., demonstrated that a daily or weekly oral intake of vitamin D can provide a protective benefit against acute respiratory infections, particularly among individuals with vitamin D deficiency [67]. These findings validate the hypothesis that preservation of adequate levels of this hormone could play a role in respiratory immune homeostasis and antiviral response.

The COVID-19 pandemic further underscored the need to assess the impact of vitamin D supplementation on the handling of respiratory viral infections. Multiple observational studies identified associations between vitamin D status and clinical outcomes such as disease severity, ICU admissions and mortality among patients infected by SARS-CoV-2. For instance, our group observed inadequate levels of calcidiol in SARS-CoV-2 infected patients undergoing ICU access or death, compared to patients achieving a favorable clinical outcome [68]. Similarly, a meta-analysis including 26 studies and more than 8000 participants demonstrated a strong association between vitamin D deficiency and increased COVID-19 severity [69]. However, this research cannot rule out reverse causality, meaning the possibility that the disease itself may contribute to reduced vitamin D concentrations. Therefore, the hypothesis that low vitamin D levels may be a consequence of the disease rather than a cause remains valid.

Currently, based on the pathophysiological considerations previously described, several interventional studies have investigated the effects of vitamin D supplementation in patients with COVID-19. Castillo et al. reported that calcidiol supplementation in addition to standard treatments was associated with reduced ICU admission of SARS-CoV-2 infected patients [73]. However, not all interventional studies highlighted beneficial effects of vitamin D supplementation. For instance, Murai et al. described that high doses of vitamin D do not diminish length of hospitalization, ICU admission and mortality in hospitalized COVID-19 patients [74]. Result heterogeneity among different studies may rely on the distinct baseline vitamin D levels, number of recruited patients, timing of supplementation, dosage and disease severity. Indeed, available data generally suggest that vitamin D supplementation may be more effective in deficient subjects and during the early stages of infection [67,75], and it may contribute to viral clearance and reduction of inflammation [76].

Besides SARS-CoV-2, vitamin D has been investigated in other respiratory infections, including RSV and influenza virus. Belderbos et al. demonstrated that low cord blood vitamin D concentrations are related with augmented risk of RSV lower respiratory tract infections during infancy [70]. Similarly, it has been described that vitamin D levels are negatively associated with RSV infection severity in infants [71]. Mechanistically, vitamin D may impact RSV infection by modulating barrier disruption. Indeed, Gao et al. reported that calcitriol treatment of airway epithelial cells prior to RSV infection induces PKA activation and protect against the increased barrier permeability and cellular dysfunction mediated by RSV [79].

Vitamin D has also been explored in influenza virus infection. In a randomized controlled trial, Urashima et al. demonstrated that supplementation of vitamin D significantly reduces influenza A in children, even though no differences have been found for influenza B [77]. However, subsequent research showed less consistent results, suggesting that the supposed protective effects of vitamin D may also depend on baseline vitamin D status, dosage and timing of supplementation. For instance, another study reported that influenza A infection occurrence was significantly reduced in the first month of supplementation, but overall vitamin D failed to decrease influenza A infection rate [78]. However, a meta-analysis including 10 RCTs highlights the role of vitamin D supplementation in decreasing the risk of this specific infection [72]. Nevertheless, clinical evidence regarding vitamin D supplementation in influenza prevention remains partially controversial.

Overall, current findings indicate that vitamin D deficiency is linked to augmented susceptibility and worse clinical outcomes in respiratory viral infections. Although some investigations suggest that vitamin D supplementation may have a potential benefit in vitamin D deficient subjects to manage the initial stages of infection and restore vitamin D homeostasis, the heterogeneity especially among interventional studies prevents definitive conclusions regarding its clinical effectiveness. Therefore, further randomized clinical trials are needed to define the optimal timing, dosage and target populations for vitamin D supplementation strategies in such diseases. Additionally, research regarding the probability of reverse causality may shed light on the potential mechanisms underlying vitamin D insufficiency/deficiency and its relationship with viral infections.

#### 4.2.2. Chronic Viral Infections

Beyond acute respiratory infections, vitamin D impact has been explored also concerning chronic viral infections, including HIV, HBV, HCV and EBV (Table 3 and Table 4). These infections are defined by persistent inflammation and immune dysfunction, all processes potentially modulated by vitamin D signaling.

Vitamin D insufficiency and deficiency have been linked to HIV disease progression, increased mortality and poorer response to antiviral therapy [80], along with reduced CD4^+^ cells count and sustained immune activation [90]. These findings have been supported by interventional studies evaluating vitamin D supplementation in HIV-infected patients. In particular a RCT demonstrated that 12 weeks of vitamin D supplementation augmented CD4^+^ T cells percentage and decreased viral RNA levels in HIV-positive subjects [86]. Moreover, another study reported that 12 months of vitamin D supplementation at high dosage significantly reduced the percentage of activated CD4^+^, CD8^+^ T cells and proinflammatory monocytes in HIV-infected patients receiving antiviral therapy [87], suggesting that vitamin D may reduce immune activation, favoring a less inflammatory immune profile. Conversely, a systematic review and meta-analysis documented nonsignificant variations in CD4^+^ cells among supplemented patients and placebo group [81]. Furthermore, vitamin D deficiency has been linked to adverse clinical outcomes in HIV patients concomitantly infected with *Mtb*, suggesting vitamin D contribution in the regulation of the immune response during these chronic infections [91]. However, it remains a matter of debate whether HIV infection reduces vitamin D levels or whether low vitamin D levels are non-specific indicators of poor health [80].

Vitamin D has also been widely investigated in chronic hepatotropic viral infections. From a general perspective, insufficiency or deficiency of this factor has been extensively reported in patients with chronic liver disease, and it has been associated with susceptibility to infections and degree of hepatic dysfunction [82,92]. However, it is not yet fully established whether vitamin D plays a real causative role in infections, or it acts merely as a spectator.

For what concerns HCV it has been demonstrated that circulating calcidiol levels predict response to antiviral treatments [88]. Furthermore, supplementation with 1(OH) vitamin D_3_ decreased cytokine production in PBMC and expression of several ISGs in liver biopsies, in addition to improving response to therapy [89]. These findings have been sustained by an interventional study evaluating cholecalciferol supplementation in patients receiving anti-HCV regimens. Results demonstrated that vitamin D supplementation improved sustained antiviral response, at least in treatment-naïve patients infected by HCV genotype 1 [88]. Considering that chronic HCV infection is defined by persistent inflammation and progressive liver damage, vitamin D may potentially modulate inflammatory and fibrotic pathways involved in disease progression. However, to date, the actual clinical relevance of the supplementation with this nutrient in HCV management remains uncertain. In any case this issue is now largely of historical significance in relation to treatments based on the previous standard of care (namely, PEG-IFN ± ribavirin) as it has not been shown to offer any virological benefit with current direct-acting antiviral therapies, likely because these are already highly effective in the vast majority (≥96%) of cases.

Among the other major hepatotropic viruses, vitamin D status has also been widely investigated in HBV infections. Indeed, low serum vitamin D levels have been related to augmented HBV replication and severe liver disease. Specifically, chronic HBV infected patients with high viral burden exhibited significantly lower circulating vitamin D levels, suggesting a possible causal relationship between active viral replication and vitamin D status [83]. Supporting these findings, treatment-naïve patients achieving undetectable levels of HBV DNA with antiviral treatments exhibited significantly higher circulating vitamin D levels compared to HBV DNA positive patients, suggesting that suppression of viral replication might result in the restoration of vitamin D homeostasis [84]. Additionally, circulating vitamin D levels have been negatively correlated with the expression of T cell exhaustion markers in HBV-infected patients, while in vitro studies have shown that calcitriol treatment promotes IFN-β expression, suggesting a potential role of this hormone also in the modulation of the antiviral response in chronic HBV infections [85]. Another proposed immunomodulatory mechanism relies on the ability of calcitriol to suppress the activity of the HBV core promoter in hepatic cell lines, thereby reducing production of HBV proteins and limiting viral transcriptional activity; in this regard there is also emerging in vivo research suggesting that the correction of vitamin D deficiency may assist patients in achieving a functional cure (e.g., loss of hepatitis B surface antigen) alongside standard antiviral treatments [93]. Taking all these considerations into account, it must be said that currently vitamin D supplementation is considered only a possible supportive, complementary strategy rather than a standalone cure for HBV, and no clinical guideline formally recommends its usage, regardless of having low blood levels.

Beyond hepatotropic viruses and HIV infections, increasing evidence suggests that vitamin D may influence host immune response to latent viruses, such as EBV, a herpesvirus that establishes latency in B lymphocytes and has been linked to the development of multiple sclerosis. As immune control against EBV mainly relies on cytotoxic T cell response, impaired vitamin D signaling may contribute to inadequate immune control and viral reactivation [94]. Indeed, low vitamin D levels are associated with low levels of CD4^+^ and CD8^+^ T cells, therefore direct EBV-infected cell killing may be impaired [95]. Moreover, it has been demonstrated that vitamin D supplementation decreases anti-Epstein–Barr Virus Nuclear Antigen-1 (EBNA-1) Immunoglobulin G (IgG) levels, however it does not increase the cytotoxic CD8^+^ T cell response against the virus [96]. Despite these findings, evidence regarding vitamin D supplementation in EBV infection remains limited, and in any case, it did not demonstrate a real clinical significance. Therefore, further experimental and interventional studies are needed to clarify whether treatment of hypovitaminosis D may improve immune control of EBV latency and reduce EBV-associated immune dysregulation.

Overall, current findings suggest that vitamin D deficiency is associated with augmented susceptibility to both acute and chronic viral infections, as well as an impaired antiviral response, persistent inflammation and poor clinical outcomes. However, the evidence regarding the direct contribution of vitamin D deficiency on disease pathogenesis does not exclude the possibility of this status to reflect the severity of the underlying disorder. Indeed, several factors may contribute to lower circulating vitamin D levels, introducing the possibility that vitamin D deficiency may be a potential marker of disease severity rather than its cause. This may explain the discrepancy between observational studies and clinical trials. More in detail, while several experimental studies suggest promising functions of vitamin D supplementation, the results of most interventional studies are heterogeneous and, in many cases, inconclusive. Importantly, several of the mechanisms modulated by vitamin D during viral infections—including dysregulated cytokine production, oxidative stress, endothelial dysfunction, impaired IFN signaling and immune exhaustion—are also major players of the progression from localized viral infection to sepsis, which is characterized by systemic inflammatory dysregulation and organ damage. The pathogenesis of these processes will be detailed in the following sections.

## 5. Sepsis: From Viral Infection to Systemic Immune Dysregulation

### 5.1. Definition and Clinical Classification of Sepsis

The progression from viral infection to sepsis represents the extreme consequence of immune dysregulation, endothelial dysfunction, and metabolic failure. Back in 1991, the Consensus Conference Committee of American College of Chest Physicians/Society of Critical Care Medicine (CHEST/SCCM) established the definition of sepsis, classified as a systemic inflammatory response syndrome (SIRS) accompanied by confirmed or suspected infection. Moreover, severe sepsis was classified as sepsis complicated by organ dysfunction, hypoperfusion or hypotension, while septic shock was described as sepsis with persistent severe hypotension not ameliorated by sufficient fluid resuscitation. Thus, initially, sepsis diagnosis was established based on the presence of infection in conjunction with at least two of the SIRS criteria [97], which included:

- Temperature > 38 °C or <36 °C;
- Heart rate > 90/min;
- Respiratory rate > 20/min or partial pressure of carbon dioxide (PaCO_2_) < 32 mmHg;
- White blood cell count > 12,000/mm^3^ or <4000/mm^3^ or >10% immature bands.

However, sepsis is a complex syndrome, characterized by several sign and symptoms, and SIRS diagnostic criteria do not provide sensitive and specific indications regarding its recognition and diagnosis, since they focus mainly on signs of systemic inflammation, which represent a host response to both infection and other insults [98]. In 2016, the Third International Consensus Definitions for Sepsis and Septic Shock (Sepsis-3) described sepsis as a life-threatening organ dysfunction due to a dysregulated host response to infection, and septic shock as sepsis requiring vasopressor therapy to elevate mean arterial pressure (MAP) to 65 mmHg or greater and serum lactate > 2 mmol/L (18 mg/dL) despite sufficient fluid resuscitation [1].

Commonly used to identify disease severity is SOFA score, which includes parameters evaluating the grade of dysfunction in the following key organs: respiratory tract, liver, cardiovascular system, kidney, central nervous system, and coagulation system. Organ failure can be assumed in presence of SOFA score ≥ 2, which is related to an increased risk of mortality. A simplified and more rapid version of SOFA is represented by the quick SOFA (qSOFA), based exclusively on respiratory frequency, systolic arterial pressure and Glasgow Coma Scale (GCS) score [99]. The importance of correctly (and promptly) diagnose and assess the severity of sepsis stems from the fact that, according to World Health Organization (WHO), this syndrome is still one of the most frequent causes of mortality, accounting for 20% of all global deaths in 2020 [100]. Among the risk factors of sepsis, and with particular reference to the infection risk, old or very young age, a weakened immune system because of diseases or medications, hospitalization ± admission to ICU, and chronic diseases are considered to play a particularly significant role [101].

### 5.2. Viral Sepsis: Definition, Diagnosis, and Clinical Relevance

Sepsis is usually caused by bacterial infections, which account for almost 90% of the cases; however, viruses, parasites and fungi may also be involved. Therefore, a precise and quick pathogen-specific recognition in the context of infection is essential to define the optimal treatment for each patient [2]. As for other microorganisms, viral infection can be identified by several clinical symptoms in addition to positive results to various microbiological tests (e.g., molecular assays, antigen detection tests or serology). A consensus regarding the specific definition of viral sepsis has not yet been reached, however most experts agree that it can be reasonably considered in the presence of signs or symptoms of sepsis not caused by bacterial, fungi or parasitic infections [102]. In particular, viral sepsis is often reported as a complication of community-acquired pneumonia (CAP). In a retrospective observational study performed in 2019 on 225 patients with CAP of viral origin—among which IAV was the most frequent infecting pathogen, followed by rhinovirus, RSV, parainfluenza virus, adenovirus, influenza B virus and coronavirus—at the time of admission sepsis was reported in 138 subjects and septic shock in 9 individuals [103]. Recently, the pandemic caused by SARS-CoV-2 has highlighted that severe COVID-19 presents the hallmarks of sepsis, including dysregulated immune response, cytokine storm, and multi-organ dysfunction [104].

Taking into account these considerations, in the following sections we will describe the immunopathogenesis of sepsis, focusing on sepsis of viral origin.

### 5.3. Immunopathogenesis of Viral Sepsis

Viral sepsis pathogenesis is complex. Similar to sepsis caused by other factors, it involves cellular and molecular mechanisms ascribable to altered immune function and imbalanced inflammatory response to a viral infection. Indeed, after pathogen recognition the hyper-inflammatory response typical of the acute phases of this condition (the so called “cytokine storm”) can be followed by, or can be concomitant to, microvascular thrombosis and profound immune suppression, which in turn lead to disruption of metabolic pathways essential for cellular function, including glycolysis, fatty acid oxidation and oxidative phosphorylation [105]. These unbalanced inflammatory response and metabolic alterations are then responsible for multi-organ damage, known as multi-organ dysfunction syndrome (MODS), which ultimately results in multiple organ failure (MOF). This is a major hallmark of all cases of sepsis, describing an acute and potentially reversible dysfunction of two or more organ systems which constitutes the most frequent cause of death in critically ill patients [106].

For what concerns viral recognition it is important to note that, unlike bacteria, viruses trigger viral sepsis primarily by exposing their nucleic acids (e.g., ssRNA, dsDNA) to intracellular PRRs, such as TLRs, RLRs, and NLRs, in a manner similar—although predictably more severe—to what was extensively described in Section 3.1 and Figure 1 about non-septic viral infections. This binding in turn activates immune cells. In this respect, the first line of defense is represented by innate immunity (including macrophages, dendritic cells, and neutrophils) that is responsible for pathogen clearance and induction of inflammation. The process is multistep and characterized by immune cells recruitment in the infection site and prompt release of pro-inflammatory cytokines like type I IFNs. In the more severe cases of sepsis, where the initial response fails to contain the virus, the same mechanisms can occasionally lead to an overzealous release of pro-inflammatory cytokines, such as IL-6, IL-1β, TNF-α and monocytic chemotactic protein (MCP), which are responsible for the aforementioned hyperinflammatory response [107]. This state changes mitochondrial permeability, increasing ROS production and therefore oxidative stress, and results in mitochondrial DNA damage and membrane alteration, with further evolution to organ damage [108]. In turn, the induction of the innate immune response activates several other molecular pathways responsible for triggering inflammation, cell metabolism, and adaptive immunity. In the ultimate end this massive surge causes bystander damage to the host’s own endothelial and epithelial cells, contributing significantly, for instance, to the life-threatening acute respiratory distress syndrome (ARDS). The direct viral invasion and cytokine storm can also activate the vascular endothelium itself, shifting it from an anticoagulant state to a pro-coagulant one. Consequently, complement system activation and platelet aggregation will result in widespread microvascular thrombosis, disrupting blood flow and causing ischemic injury to vital organs [101].

At a later stage of the course of sepsis, to prevent further fatal hyperinflammation and prolonged inflammatory states, the body starts to mount a compensatory anti-inflammatory response (CARS). This is a physiological reaction; however, in viral sepsis it is often disproportionate. This complex process consists in the downregulation of surface molecules involved in immune cell activation, upregulation of immune exhaustion markers, such as PD-1/programmed death-ligand 1 (PD-L1), immune cell apoptosis (e.g., of B and T-lymphocytes), and T cell exhaustion. The functional consequence of all this can be a profound suppression of the adaptive immune system, rendering patients highly susceptible to secondary bacterial and fungal infections [109].

## 6. Vitamin D in the Context of Sepsis: Does It Also Play a Role in Viral Forms?

Given the pleiotropic immunomodulatory functions of vitamin D, it has been hypothesized that vitamin D status may influence many of the above-described key pathogenic pathways involved in sepsis, including viral-induced types [110]. The fact remains, however, that, to date, the vast majority of experimental and interventional studies—whether in animal models or in patients—refer, to the best of our knowledge, only to the forms of sepsis with bacterial etiology; a few investigations do not even specify the exact etiology of the sepsis itself, but it is reasonable to assume that they too fall within the category described above. Therefore, direct evidence regarding the role of vitamin D in specific context of viral sepsis is rather scarce [111].

Relevant exceptions to this are represented by adulthood respiratory viruses (e.g., influenza viruses) [112,113,114,115] and, most importantly, by SARS-CoV-2 when causing the most severe forms of COVID-19 [116,117,118]. There is also some evidence regarding the role of vitamin D—both in prevention and treatment—in viral sepsis during pregnancy and in the pediatric age group. Specifically, vitamin D deficiency in pregnant women and in umbilical cord blood has been identified as a significant risk factor for severe RSV infections in the first year of life and early childhood [70,119,120,121]. In this respect, the literature has long focused on the link between vitamin D and child respiratory health in general, suggesting a possible protective role on lungs, starting from the prenatal period, through its known effects on cellular immunity with reduction of oxidative stress and airway remodeling. It is therefore currently considered plausible that, at least in these age groups, this nutrient, particularly in deficient subjects, can reduce the burden of respiratory viral sepsis, including those of lower tract and attributable to pneumonia and bronchiolitis. This beneficial effect does not seem to consist solely in preventing the infectious events themselves, but, above all, in mitigating the disease severity in those who develop acute episodes and progress to a septic state (e.g., concerning pneumonia, favoring shorter duration of resolution, lower need for ICU admission, and/or shorter hospital/ICU stay), although a significant effect on overall mortality has generally not been demonstrated [77,122,123,124,125,126].

Coming back to the adult and elderly population, as above reported, the specific available evidence on viral sepsis is rather limited and centered mainly on bacterial forms. While bearing in mind that this may introduce a clear bias in the interpretation of the available literature at least as far as these age groups are concerned, from a broader perspective it is still evident that several observational studies and meta-analyses have demonstrated a relationship between vitamin D deficiency and greater probability of sepsis, increased disease severity, extended hospital stay and higher mortality [127,128,129,130,131,132].

However, interventional studies analyzing vitamin D supplementation in septic patients reported heterogeneous results. Specifically, it has been reported that vitamin D supplementation in these subjects reduces circulating pro-inflammatory cytokines and increases vitamin D bioavailability, which is related to increased defensin and cathelicidin production [133,134,135,136]. Additionally, findings indicated that vitamin D supplementation could mitigate disease severity and ameliorate clinical outcomes in these patients [112,137,138,139,140]. Concerning the specific COVID-19 sepsis, these anti-inflammatory mechanisms could also reside in this hormone-induced counterbalancing of the renin-angiotensin system network. Vitamin D can indeed induce the expression of angiotensin-converting enzyme 2—the fusion receptor of the virus—which in turn causes a marked reduction in the inflammatory response at both the systemic and pulmonary levels through a complex regulation of the immune system [133]. Nonetheless, other investigations failed to demonstrate a reduction in mortality, length of hospital stay, or ICU admissions following vitamin D supplementation to septic patients [141,142,143]. For the sake of completeness, it should be noted that even a minority of studies specifically focused on children have yielded inconclusive results concerning the same outcomes, particularly when influenza or RSV were not considered [78,144,145,146,147,148] Therefore, whether vitamin D supplementation may exert clinically relevant effects during viral sepsis remains controversial: unreliability in this evidence is mainly due to imprecision, risk of bias, inconsistency, and indirectness [127,149].

To try to address all these uncertainties, it is important to consider that so many pathways regulated by vitamin D overlay with the pathogenesis of sepsis. Exploring the immunological roles of vitamin D in sepsis could thus help to better clarify this disease pathophysiology. For instance, it has long been well known that hyperactivation of NF-κB signaling and excessive production of pro-inflammatory cytokines contribute to the hyperinflammatory response characteristic of the early phases of sepsis. In this respect, vitamin D negatively regulates NF-κB thereby dampening pro-inflammatory pathways [7], as for instance demonstrated in some forms of COVID-related sepsis [133]. Therefore, it is reasonable to speculate that impaired vitamin D signaling may favor uncontrolled cytokine production and amplification of systemic inflammation [150].

Focusing on the immunomodulation of respiratory viruses, there is also a known genetic susceptibility predominantly associated with innate immune genes which, as previously mentioned, are in turn modulated by VDR. In this regard, a reasonable—although not yet validated—strategy for identifying high-risk patient subgroups could consist in the analysis of specific single-nucleotide polymorphisms (SNPs) [151]. To the best of our knowledge, this has been investigated so far only for RSV bronchiolitis-associated sepsis in children. Specifically, a large-scale meta-analysis reportedly demonstrated a statistically significant association between FokI (rs2228570)—a specific SNP in the start codon of the VDR gene—and the development of the most severe forms of RSV [152].

Besides hyperinflammation, advanced phases of sepsis are defined by immune suppression, comprising lymphocyte apoptosis, flawed antigen presentation and T cell exhaustion. Given the ability of vitamin D to modulate both innate and adaptive immune responses, dysregulated vitamin D signaling may contribute not only to hyperinflammation, but probably also to the immune dysfunction characteristic of the late stages of this continuous process [153,154]. Similarly, an altered vitamin D (and gut microbiota) homeostasis could also worsen the immune-senescence and inflammaging typical of the frail elderly with co-existing medical diseases, affecting the severity and mortality of viral sepsis episodes such as in the most severe forms of COVID-19 [155].

Another major hallmark of sepsis consists in endothelial dysfunction and disruption of vascular integrity, which lead to increased permeability, hypotension and microvascular thrombosis (as reported in Section 5.3). As a result, these alterations may contribute to impaired tissue perfusion, organ dysfunction and, ultimately, increased mortality. These phenomena have been demonstrated in all forms of sepsis, including specifically those of viral origin, at least for what concerns SARS-CoV-2 infections. In this regard, it has been suggested that vitamin D deficiency promotes a prothrombotic state and that, conversely, vitamin D reduces platelet aggregation through the expression modulation of plasminogen activator inhibitor-1, thrombospondin-1 and thrombomodulin [156,157,158,159]. Thus, vitamin D analogs may potentially protect against the formation of platelet–leukocyte aggregates responsible for exacerbating MODS in sepsis [160]. As a matter of fact, it has been shown that, at least in mouse models, supplementation with calcitriol appears to improve sepsis-induced coagulation disorders, although no effects on platelet function were observed in the LPS-treated animals [161].

Altogether, these findings do indicate that vitamin D may have an impact on several molecular and immunological pathways contributing to the pathogenesis of various forms of sepsis—including those of viral origin—albeit further experimental and interventional studies are still required to clarify its real clinical significance in critically ill patients [162,163,164].

## 7. Conclusions

Vitamin D is increasingly recognized as an important immunomodulator involved in the regulation of antiviral immunity and systemic inflammatory responses. Beyond its classical role in calcium and bone homeostasis, emerging findings demonstrated that it can modulate also innate and adaptive immune responses throughout the regulation of inflammation, antimicrobial peptide production, autophagy and oxidative stress. These pathways are particularly relevant in host defense against viral pathogens, and in the progression from focal infection to systemic immune dysregulation, providing the biological rationale for the interplay between vitamin D and viral infections.

However, the strength of available research differs substantially according to the evidence considered. Experimental studies showed that vitamin D signaling interacts with several pathways involved in inflammation and tissue injury, therefore proposing a potential role of this vitamin in antiviral response and sepsis pathogenesis. These mechanisms may be relevant in severe respiratory viral infections and viral sepsis (as in many COVID-19 cases), where dysregulated immune activation and endothelial injury drive organ dysfunction and worse clinical outcomes. Moreover, several observational investigations showed an association between low vitamin D levels and augmented susceptibility to both acute and chronic viral infections, along with higher disease severity and fatality rate. However, this correlation does not establish causality and may be influenced by disease severity and other confounding factors. In addition, interventional studies exploring vitamin D supplementation were, for the most part, quite heterogeneous and, often, inconclusive.

Therefore, the exact clinical relevance of vitamin D supplementation in viral infections and/or viral sepsis remains unclear. Future research should address whether specific populations, particularly those with vitamin D deficiency, may clinically benefit from its administration. Additionally, further studies should clarify the optimal dose and timing, while also addressing the potential link between vitamin D signaling and immune/metabolic dysfunction during severe viral diseases.

## Figures and Tables

**Table 1:** Summary of observational studies and meta-analyses on vitamin D in the context of respiratory viral infections. Details on supplementation regimens and methodologies of the studies are summarized in the footnotes.

Virus	Study Design	Study Population	Primary Outcome	Main Findings	Limitations
Acute respiratory tract infections [67]	Meta-analysis of randomized controlled trials (RCTs) ^1^	10,933 participants from 25 RCTs	At least one acute respiratory tract infection (ARI).	Reduction of ARI. Greatest benefits in patients with vitamin D deficiency, particularly with daily or weekly oral regimen, whereas no protective effects of bolus administration were observed.	Heterogeneity between trials regarding vitamin D baseline levels, treatment regiment, timing and study populations.
SARS-CoV-2 [68]	Prospective observational cohort study	139 SARS-CoV-2 patients	Association between vitamin D levels and disease outcome.	Higher baseline vitamin D levels independently predicted favorable outcome. Vitamin D > 11.1 ng/mL predicted a positive clinical outcome.	Single center study, small sample size; the observational nature of the study precluded causal inference.
SARS-CoV-2 [69]	Meta-analysis	8176 patients from 26 studies	Association between vitamin D deficiency and disease severity.	Vitamin D deficiency was associated with increased disease severity. No association with disease risk was found.	No sex stratification, no confounding factors evaluated; methodological differences within studies.
RSV [70]	Prospective cohort study	156 infants	Association between vitamin D levels at birth and risk of RSV infection in the first year.	Low vitamin D concentrations at birth were associated with the risk of RSV infection.	Low sample size, lack of information on sunlight exposure and vitamin D dietary intake.
RSV [71]	Prospective cohort study	125 infants hospitalized for RSV infection	Association between vitamin D levels at admission and RSV disease severity.	Patients with worst disease had lower vitamin D levels. Vitamin D deficiency was a risk factor for RSV infection.	Single center study, vitamin D intake data were obtained retrospectively.
Influenza virus [72]	Meta-analysis of RCTs ^2^	8029 patients from 10 RCTs	Effects of vitamin D supplementation on the risk of influenza infection.	Vitamin D supplementation reduced the infection risk.	Absence of vitamin D baseline levels in some studies, wide age range.

^1^ Meta-analysis of randomized, double-blind, placebo-controlled trials on cholecalciferol or ergocalciferol supplementation of any duration. ^2^ All studies used cholecalciferol as supplementation, and control group received either placebo or low dose cholecalciferol, i.e., 400 international units (IU)/day.

**Table 2:** Summary of interventional studies regarding the effects of vitamin D supplementation in the context of respiratory viral infections. Details on supplementation regimens and methodologies of the studies are summarized in the footnotes.

Virus	Trial Design	Study Population	Treatment	Primary Outcomes	Main Findings	Limitations
SARS-CoV-2	Randomized open label, double masked [73]	76 SARS-CoV-2 patients	Standard care ± calcidiol ^1^	Rate of ICU admission and death.	Calcidiol supplementation reduced the need of ICU admission.	Small sample size; absence of placebo group, baseline vitamin D status and comparison with cholecalciferol.
SARS-CoV-2	Double-blind, randomized, placebo- controlled [74]	237 SARS-CoV-2 patients	Oral cholecalciferol or placebo ^2^	Length of hospitalization.	Cholecalciferol supplementation did not influence the length of hospital stay.	Small sample size, interference of concomitant medications, low number of patients with vitamin D deficiency.
SARS-CoV-2	Multicenter, open-label, parallel group, randomized controlled [75]	253 SARS-CoV-2 patients	Oral cholecalciferol ^3^	Mortality within 14 days.	Cholecalciferol administration within 72 h after SARS-CoV-2 infection reduced 14-days mortality independently of vitamin D baseline levels.	Risk of bias due to the open-label design, absence of placebo
SARS-CoV-2	Randomized, placebo-controlled [76]	40 SARS-CoV-2 patients with vitamin D deficiency	Oral cholecalciferol or placebo ^4^	Effects of supplementation on viral clearance.	Increased SARS-CoV-2 negativity within 21 days in supplemented patients.	Only asymptomatic or mildly symptomatic patients considered; high cholecalciferol dose.
Influenza virus	Randomized, double blind, placebo controlled [77]	430 pediatric healthy individuals	Oral cholecalciferol or placebo ^5^	IAV incidence.	Decreased incidence of IAV between 31–60 days of supplementation.	Small sample size, vitamin D supplementation outside the study drug not prohibited, absence of reported vitamin D baseline levels.
Influenza virus	Randomized, double blind, placebo controlled [78]	247 young healthy volunteers.	Oral cholecalciferol or placebo ^6^	IAV incidence.	Short term supplementation (1 month) decreased the incidence of IAV infection. 2 month supplementation did not influence overall incidence.	Absence of vitamin D baseline levels, small sample size.

^1^ Patients were randomized to receive or not receive calcidiol in addition to standard care in a 2:1 ratio. Subjects in the calcidiol group received 0.532 mg oral calcidiol on day of admission, followed by 0.266 mg oral calcidiol on days 3 and 7 and then weekly until discharge or intensive care unit (ICU) admission. Standard care consisted of hydroxychloroquine, azithromycin and broad-spectrum antibiotics, when required. ^2^ Patient were randomized 1:1 to receive a single dose of either 200,000 IU oral cholecalciferol or placebo. ^3^ Patients were randomized 1:1 to receive a single dose of either 40,000 IU or 50,000 IU of cholecalciferol. ^4^ Subjects were randomized to receive a single dose of either 60,000 IU oral cholecalciferol or placebo. Vitamin D levels were measured at 7 days. In the interventional group, patients reaching vitamin D > 50 ng/mL were administered a single dose of 60,000 IU cholecalciferol, while patients with vitamin D < 50 ng/mL were administered with 60,000 IU cholecalciferol/day until day 14. ^5^ Patients received 1200 IU/day oral cholecalciferol for 90 days. ^6^ Subjects received 2000 IU oral cholecalciferol/day for 2 months. IAV diagnosis was performed through rapid test.

**Table 3:** Summary of observational studies and meta-analyses regarding vitamin D in the context of chronic viral infections. Further details of these studies are summarized in the footnotes.

Virus	Study Design	Study Population	Primary Outcome	Main Findings	Limitations
HIV	Case-cohort study [80]	250 treatment-naïve HIV patients initiating combination antiretroviral therapy (cART)	HIV progression, death, treatment response.	Patients with low vitamin D levels had higher risk of disease progression, death and virological failure.	Exclusion of patients with major comorbidities or laboratory alterations, no data on vitamin D supplementation.
HIV	Meta-analysis [81]	966 young participants from 10 RCTs ^1^	Vitamin D and PTH levels, bone mineral density and CD4^+^ cells percentage.	Increased vitamin D serum concentrations. No effects reported for PTH levels, bone mineral density and CD4^+^ T cells percentage compared to placebo.	No data on antiviral therapy, short study duration, heterogeneity among studies regarding intervention, timing, age and HIV stages.
HCV	Prospective observational cohort study [82]	291 patients with HCV-related cirrhosis ^2^	Susceptibility to infections and prevalence of vitamin D deficiency.	Vitamin D deficiency was associated with the presence of infection and could predict the development of new infections in the three-month follow-up.	Single center study, causality could not be assessed.
HBV	Retrospective observational study [83]	203 treatment-naïve HBV infected patients	Association between HBV and vitamin D levels.	Low levels of vitamin D were associated with high HBV replication.	Association could not prove causality, low median stage of liver fibrosis, small sample size.
HBV	Prospective observational cohort study [84]	128 treatment-naïve HBV infected patients and 128 healthy controls	Relationship between vitamin D levels and HBV parameters.	HBV treatment-naïve patients had lower vitamin D levels than controls, and these levels improved after antiviral treatment.	Observational design, small sample size.
HBV	Prospective observational study with complementary in vitro analyses [85]	86 HBV-infected patients ^3^	Association between vitamin D levels, T cell exhaustion and IFN-β expression.	Lower vitamin D levels were associated with increased T cell exhaustion markers. In vitro, calcitriol reduced T cell exhaustion and promoted IFN-β expression.	In vitro findings may not translate into clinical benefits.

^1^ Randomized controlled trials supplementing patients with cholecalciferol, ergocalciferol or placebo. ^2^ At baseline, vitamin D levels were quantified using a chemiluminescence immunoassay. Patients were then monitored over a three-month period for the onset of any infection. Infection at baseline was present in 21.9% of the studied population. ^3^ Enrolled patients had different stages of liver disease: chronic hepatitis, cirrhosis or hepatocellular carcinoma.

**Table 4:** Summary of interventional studies regarding the effects of vitamin D supplementation in the context of chronic viral infections. Details on supplementation regimens and methodologies of the studies are summarized in the footnotes.

Virus	Trial Design	Study Population	Treatment	Primary Outcome	Main Findings	Limitations
HIV	Randomized double-blind trial [86]	44 patients	Cholecalciferol ^1^.	Immunological and virological status.	Vitamin D supplementation induced a small increase in CD4^+^ T cell count, and a slight decrease in viral load.	Small sample size, absence of placebo group.
HIV	Randomized, active-control, double-blind [87]	51 young patients on stable cART	Cholecalciferol ^2^.	Alterations in immune activation and exhaustion markers.	The high dose cholecalciferol group showed decreased percentages of activated CD4^+^, CD8^+^ cells and monocytes.	Absence of adherence data and placebo group, small sample size.
HCV	Intention-to-treat prospective randomized [88]	72 treatment-naïve HCV genotype 1 infected patients	Cholecalciferol ^3^.	Response to antiviral therapy.	Vitamin D baseline levels and supplementation were predictors of sustained virological response.	Small sample size, lack of reported vitamin D levels during treatment, absence of placebo, limited applicability to current antiviral therapies.
HCV	Multi-center, prospective case-control trial [89]	84 HCV genotype 1b cirrhotic patients	1 (OH) vitamin D_3_ ^4^.	Immunological response following supplementation.	Supplementation augmented immune response and enhanced virological response to therapy.	Non-randomized case-controlled study, small sample size, limited applicability to current antiviral therapies.

^1^ Patients were randomized to receive either 4000 IU or 7000 IU oral cholecalciferol/day for 12 weeks. Viral load was assessed by quantitative assay at baseline, 6 weeks and 12 weeks, while the immunological landscape was detected by flow cytometry. ^2^ Patients were randomized to receive either 18,000 IU/month (standard dose), 60,000 IU/month (moderate dose) or 120,000 IU/month (high dose). Monocytes, CD4^+^ and CD8^+^ T cells, along with exhaustion markers, were assessed by flow cytometry. ^3^ Patients were randomized to receive standard therapy (pegylated (PEG)-IFN-α-2b + ribavirin) with or without 2000 IU/day of cholecalciferol. Cholecalciferol treatment started 4 weeks before initial of antiviral treatment and continued up to 48 weeks. ^4^ Patients were divided into treatment (N = 42) and control group (N = 42). The treatment group included in turn two subgroups. In the first one, 18 patients were supplemented with 1 μg/day of 1(OH) vitamin D_3_ for 4 weeks before the initiation of standard HCV therapy (PEG-IFN-α-2b + ribavirin) and for the following 24 months of treatment; blood samples and PBMCs were collected at −4 week, week 0, week 4, week 12 and week 24. The second part of the treatment group included 24 patients who were supplemented with 1 μg/day of 1 (OH) vitamin D_3_ in concomitance of the initiation of standard HCV therapy; samples were collected at week 0, week 4, week 12 and week 24. The control group consisted of matched historical subjects treated with PEG-IFN-α/ribavirin; samples were collected at −4 week, week 0, week 4, week 12 and week 24.

## Data Availability

No new data were created or analyzed in this study. Data sharing is not applicable to this article.

## Abbreviations

The following abbreviations are used in this manuscript:

A549	Adenocarcinoma Human Alveolar Basal Epithelial Cells
ACE2	Angiotensin-converting enzyme-2
AIM2	Absent in Melanoma 2
AP-1	Activator Protein-1
APCs	Antigen Presenting Cells
ARDS	Acute Respiratory Distress Syndrome
ARI	Acute Respiratory Tract Infection
ATG7	Autophagy Related 7
ATP	Adenosine Triphosphate
BACH2	BTB Domain and CNC Homolog 2
BECN1	Beclin 1
CAP	Community-Acquired Pneumonia
CARS	Compensatory Anti-Inflammatory Response
cART	Combination Antiretroviral Therapy
CD	Cluster Differentiation
CEBPB	CCAAT Enhancer Binding Protein Beta
c-JUN	Jun Proto-oncogene, AP-1 Transcription Factor Subunit
cGAMP	Cyclic GMP-AMP
cGAS	Cyclic Guanosine Monophosphate Adenosine Monophosphate Synthase
CHEST	American College of Chest Physicians
CLRs	C-type Lectin Receptors
COVID-19	Coronavirus Disease 19
CpG	Cytosine-phosphate-Guanine
CTLA-4	Cytotoxic T Lymphocyte Associated Protein-4
CXCL	C-X-C Motif Chemokine Ligand
DAMPs	Damage-Associated Molecular Patterns
DCs	Dendritic Cells
defB2	Defensin-β2
dsDNA	Double Strand DNA
dsRNA	Double Strand RNA
EBNA-1	Epstein–Barr Virus Nuclear Antigen-1
EBV	Epstein–Barr virus
GCS	Glasgow Coma Scale
GMP-AMP	Guanosine Monophosphate-Adenosine Monophosphate
HAMP	Hepcidin Antibacterial Protein
HBV	Hepatitis B Virus
HCV	Hepatitis C Virus
HIV	Human Immunodeficiency Virus
HSV	Herpes-Simplex Virus
IAV	Influenza A virus
ICU	Intensive Care Unit
IFI16	Interferon Gamma Inducible Protein 16
IFNAR	IFNα Receptor
IFN	Interferon
IgG	Immunoglobulin G
IKK	Inhibitory κB Kinase
IL	Interleukin
IRF	Interferon Regulatory Factor
ISG15	IFN-stimulated Protein of 15 kDa
ISGF3	IFN-stimulated Gene Factor 3
ISGs	IFN-stimulated Genes
ISREs	IFN-stimulated Response Elements
IU	International Units
IκBα	Nuclear Factor of Kappa Light Polypeptide Gene Enhancer in B-cells Inhibitor, Alpha
JAK1	Janus Kinase 1
kDa	Kilodalton
LAG-3	Lymphocyte Activation Gene 3
LC3B	Microtubule-associated Protein 1 Light Chain 3 Beta
LILRB4	Leukocyte Immunoglobulin Like Receptor B4
LL37	Cathelicidin
LPS	Lipopolysaccharide
MAP	Mean Arterial Pressure
MAPK	Mitogen-Activated Protein Kinases
MAVS	Mitochondrial Antiviral-Signaling Protein
MCP	Monocytic Chemotactic Protein
MDA5	Melanoma Differentiation-Associated Gene 5
MDM	Monocyte-Derived Macrophages
MDP	Muramyl Dipeptide
MHC	Major Histocompatibility Complex
MODS	Multi-Organ Dysfunction Syndrome
MOF	Multiple Organ Failure
*Mtb*	*Mycobacterium Tuberculosis*
Mx	Myxovirus Resistance
MyD88	Myeloid Differentiation Primary Response Protein 88
Nf-E2	Nuclear Factor Erythroid-2
NF-κB	Nuclear Factor Kappa-light-chain-enhancer of Activated B Cells
NFAT	Nuclear Factor of Activated T-cells
NK	Natural Killer
NLRs	NOD-Like Receptors
NOD	Nucleotide-Binding and Oligomerization Domain
Nrf2	Nf-E2-Related Factor 2
OAS	2′,5′-oligoadenylate Synthetase
PaCO_2_	Partial Pressure of Carbon Dioxide
PAMPs	Pathogens-Associated Molecular Patterns
PBMCs	Peripheral Blood Mononuclear Cells
PD-1	Programmed Cell Death-1
PD-L1	Programmed Death-Ligand 1
pDCs	Plasmacytoid DCs
PEG	Pegylated
PI3K	Phosphatidylinositol-3 Kinase
PKA	Protein Kinase A
PKC	Protein Kinase C
PKR	Protein Kinase R
PLA2	Phospholipase A2
PLC	Phospholipase C
PRR	Pathogen-Recognition Receptor
PTH	Parathyroid Hormone
qSOFA	Quick SOFA
RCT	Randomized Controlled Trial
RIG-I	Retinoic Acid-Inducible Gene-I
RLRs	RIG-I-Like Receptors
RNA pol III	RNA Polymerase III
RNaseL	Ribonuclease L
ROS	Reactive Oxygen Species
RSV	Respiratory Syncytial Virus
RXR	Retinoid-X-Receptor
SARS-CoV-2	Severe Acute Respiratory Syndrome Coronavirus
SCCM	Society of Critical Care Medicine
scRNA-seq	Single-cell RNA Sequencing
Sepsis-3	Third International Consensus Definitions for Sepsis and Septic Shock
SIRS	Systemic Inflammatory Response Syndrome
SNPs	Single-nucleotide Polymorphisms
SOCS	Suppressor of Cytokine Signaling
SOFA	Sequential Organ Failure Assessment
ssRNA	Single Strand RNA
STAT	Signal Transducers and Activators of Transcription
STING	Stimulator of Interferon Genes
SYK	Spleen Tyrosine Kinase
TBK	TANK-binding Kinase
TCR	T Cell Receptor
Tfh	T Follicular Helper
Th	T Helper
TIM-3	T Cell Immunoglobulin Domain and Mucin Domain 3
TIR	Toll/Interleukin-1 Receptor Domain
TLRs	Toll-Like Receptors
TNF	Tumor Necrosis Factor
Treg	T Regulatory
TREM	Triggering Receptor Expressed on Myeloid cells
TRIF	TIR domain-containing Adapter-inducing Interferon-β
TYK2	Tyrosine Kinase 2
U937	Human Promonocytic Leukemia Cells
USP18	Ubiquitin-specific protease 18
UV	Ultraviolet
VDBP	Vitamin D Binding Protein
VDR	Vitamin D Receptor
VDREs	Vitamin D Response Elements
WHO	World Health Organization
